# Experimental Infection of *Ornithodoros erraticus sensu stricto* with Two Portuguese African Swine Fever Virus Strains. Study of Factors Involved in the Dynamics of Infection in Ticks

**DOI:** 10.1371/journal.pone.0137718

**Published:** 2015-09-14

**Authors:** Rita Ribeiro, Joachim Otte, Sara Madeira, Geoff H. Hutchings, Fernando Boinas

**Affiliations:** 1 Centre for Interdisciplinary Research in Animal Health (CIISA), Faculty of Veterinary Medicine, University of Lisbon, Lisbon, Portugal; 2 FAO Regional Office for Asia and the Pacific, 39 Phra-Atit Road, 10200 Bangkok, Thailand; 3 The Pirbright Laboratory, Institute for Animal Health, Ash Road, Woking, Surrey, GU24 0NF, United Kingdom; Umeå University, SWEDEN

## Abstract

African swine fever (ASF) is a frequently devastating hemorrhagic disease of domestic pigs and wild boar and *Ornithodoros erraticus sensu stricto* argasid ticks are the only biological vectors of African swine fever virus (ASFV) known to occur in Europe. Recently this disease emerged in Eastern Europe and Russian Federation, showing a huge potential for a rapid spread between countries. There is some risk of re-emergence of ASF in the countries where these ticks exist, that can contribute for the persistence of infection and compromise control measures. In this study we aimed to identify factors that determine the probability of infection and its dynamics in the tick vector *Ornithodoros erraticus sensu stricto*, with two Portuguese strains of ASFV. Our results suggest that these ticks have a high likelihood of excreting the two haemadsorbing ASF viruses of different host origins and that, in field surveys, the analysis of adults and 5^th^ nymphal stage can provide the best chance of detecting virus infection. The results also indicate that infection of pigs with highly virulent ASF viruses will promote higher rates of infection and a higher likelihood for virus excretion by ticks. Nevertheless, there is also a risk, although lower, that ticks can become infected on pigs that have overcome the acute phase of infection, which was simulated in our study by membrane feeding ticks with low titres of virus. We believe these results can be valuable in designing and interpreting the results of ASF control programmes, and future work can also be undertaken as our dataset is released under open access, to perform studies in risk assessment for ASFV persistence in a region where *O*. *erraticus sensu stricto* ticks are present.

## Introduction

African swine fever (ASF), a frequently devastating hemorrhagic disease of domestic pigs and wild boar, is caused by African swine fever virus (ASFV), the only known DNA arbovirus and the sole member of the family Asfarviridae [1].

In affected countries the disease limits pig production, resulting in a mortality rate approaching 100% in domestic pigs [2,3], causing significant economic losses and affecting food security and results in the imposition of severe international trade restrictions [4,5]. Current control strategies rely on the combination of culling pigs and rigorous sanitary measures [6].

ASFV is very well adapted to its natural hosts and, in East and Southern Africa, is maintained in an ancient sylvatic cycle involving warthogs (*Phacochoerus africanus*) [7–9] in conjunction with *Ornithodoros porcinus* (*Ornithodoros moubata* complex) (Walton,1979) [10], a long-lived and nidicolous argasid tick [11,12].

In the Iberian Peninsula, the only biological vector of ASFV known to occur is *Ornithodoros erraticus sensu stricto*, which is found in holes, cracks and fissures in the walls of the traditional buildings used to house pigs [6, 13–15].

Taxonomic classification of this species is controversial. A recent publication [16] based on genetic studies proposes that the specimens present in Iberian Peninsula could be a new species–*O*. *occidentalis*–but confirmation studies need to be performed in order to this new taxonomical classification be accepted. In the current paper, the authors adopt the commonly used designation of the species, *O*. *erraticus sensu stricto*, based on the currently accepted systematics of ticks [17].

The biological cycle of *O*. *erraticus sensu stricto* ticks includes the eggs and the stages of larvae, nymphs (N) (3 to 5 stages) and adults (male and female). The larvae and each nymphal stage need a bloodmeal before passing to the next life cycle stage [18].

In Spain these soft ticks were recognized as an important cause of re-occurrence of ASF outbreaks [4] and, in Portugal, they were identified as the most likely cause of the last ASF outbreak that occurred in the country in 1999 [2,19]. In fact, they are important reservoirs for ASFV, being able to maintain the virus for more than five years after infection [2].

The recent spread of ASFV in the Caucasus region, Russian Federation and Eastern Europe and the presence of ticks potentially capable of acting as vectors of the disease [20] is a matter of concern. The infection of ticks could lead to a rise in the rate of persistence of the virus and difficulties in disease control [6]. Furthermore, it is known that the ASFV strain circulating in the Caucasian region, Georgia 2007/1, can replicate efficiently in *O*. *erraticus sensu stricto* ticks, obtained from pig farms in southern Portugal [3].

The recent re-emergence of ASFV in Europe makes it essential to understand which intrinsic and extrinsic factors can affect the dynamics of infection in *O*. *erraticus sensu stricto* ticks.

For a tick to be able to transmit ASFV, the virus must overcome a series of obstacles from ingestion until it can be transmitted to another host: the gut wall must be passed, the virus needs to replicate in the tick’s tissues and needs to reach the secretory glands, mainly the salivary glands (and sometimes coxal glands) where it should be present for inoculation when feeding on the new host [21].

Several tick-pig infection and transmission studies have been performed with geographically diverse soft tick species of the *Ornithodoros* genus, such as *O*. *savignyi* [22] from Africa; *O*. *coriaceus* and *O*. *turicata* [23,24] from North America, and *O*. *puertoricensis* from the Caribbean [24,25]. In all cases, transmission of ASFV has been proved [9]. In the Russian Federation and Caucasus region the knowledge of tick distribution and host preferences is limited, and ticks have not been involved in experiments to demonstrate the transmission of ASFV. However, because argasid ticks reported in these areas can be very closely related to *O*. *erraticus sensu stricto* ticks, their role as potential vectors cannot be ruled out [20].

Extensive studies with *O*. *moubata* complex as a reservoir of ASFV have been undertaken to determine the effects of ASFV infection and the ability of the ticks to maintain and transmit the virus [1, 26]. Transovarial, transtadial and sexual ASFV transmission has been described for this species [11, 27, 28]. Other experiments established that after oral infection in *O*. *moubata*, some viruses multiply in midgut cells before dissemination to other tick tissues and that there is a clearance of tick infection with time [29]. Ticks from this complex which achieved a titre of 10^4^ HAD_50_ were more likely to excrete ASFV when feeding [30]. The existence of a midgut barrier to infection was also demonstrated [31].

Previous studies on the role of *Ornithodoros erraticus* as ASFV vectors have shown that transtadial and sexual transmission can occur [18, 28] but evidence for transovarial transmission is lacking [32]. Also for these ticks the presence of a gut barrier that impairs virus replication after oral infection with low virus titres has previously been demonstrated [28, 31].

In this paper we describe a series of experimental infections of *O*. *erraticus sensu stricto* ticks with two strains of ASFV to evaluate factors that influence the dynamics of infection and which could influence the likelihood of virus excretion by ticks.

The specific objectives of the experiments were to evaluate the ability of different pathogenic strains of viruses from tick (OUR T88/1) or pig (Tomar87) origins at different titres, to infect *O*. *erraticus sensu stricto* ticks of different developmental stages by three different routes of exposure and to study the establishment of infection and the likelihood of virus excretion (LVE) by ticks. We infer about the LVE based on the virus titres found in the tick. In addition to using the oral route of infection via “in vivo” pig feeding and “in vitro” membrane feeding, virus was also inoculated directly into the haemocoel, by-passing the gut, in order to evaluate the existence of a gut barrier to the ASF virus strains studied.

## Materials and Methods

The factors tested were (i) ASFV strain (Tomar 87 and OURT88/1), (ii) the route of exposure (pig feeding, membrane feeding and inoculation), (iii) the titre of virus exposure (‘high’ and ‘low’), (iv) tick stage (adults (A), nymphs in the fifth (n5), fourth (n4), third (n3), second (n2) and first (n1) stages), (v) in adult ticks, the gender (male and female) and (vi) days post exposure (DPE).

### Ethics statement

The experiments on pigs described below were carried out at The Pirbright Institute (Pirbright Laboratory, Ash Road, Pirbright, Surrey) under Home Office Licence 90/00752. Although all experimental protocols on project licenses were formally approved by the Home Office Inspector, the experiments were carried out prior to the requirement for an Ethical Review Committee. The experimental design was retrospectively reviewed by the current Ethics Committee for Laboratory Animals at the Faculty of Veterinary Medicine (FMV) on the 7^th^ of February 2011, who indicated that in their opinion all ethical procedures were designed according to good animal practices with respect to welfare, and were carried out according to the Council Directive regarding the protection of animals used for experimental and other scientific purposes (6/609/EEC), and National Legislation.

The results presented in this article were obtained in an experiment performed in two pigs and using exactly the same protocol as mentioned in the paper “The persistence of African swine fever virus in field-infected *Ornithodoros erraticus* during the ASF Endemic Period in Portugal”, Boinas et al., 2011, published by PLOS ONE.

Permission to collect ticks in the field was formally approved by the Portuguese Official Veterinary Services. In all contacted farms the owner of the land gave permission to conduct the study.

The field study did not involve endangered or protected species. We used two domestic pigs (*Sus scrofa*) and *Ornithodoros erraticus sensu stricto* ticks.

### Tick samples

Ticks used in the experiments were collected on pig farms in the South of Portugal, Alentejo and Algarve, the regions that have been the most affected by ASFV outbreaks [2], using manual collection, CO_2_ trapping, or both [15]. The ticks originated from farms with larger tick populations, on which clinical ASF had not been reported for the previous 10 years, and where a minimum of 5% of the pigs present had been tested once a year for ASF antibodies with negative results. Ticks from these farms had been previously examined and found to be virus-free [18].

The locations where *O*. *erraticus sensu stricto* ticks used in the experiment were collected are referred in S1 Table. We provided the name of the parishes and the geographical coordinates of parish centroid where collections were performed.

A total of 499 *O*. *erraticus sensu stricto* ticks were used in this experiment. Ticks were characterized morphologically by parasitologists at the Faculty of Veterinary Medicine, Lisbon, Portugal. With respect to gender, ticks were classified by observation of the genital openings, while stage of development was determined according to their body length, using morphological characteristics with standard taxonomic keys [33, 34].

### Virus strains

Two different ASF virus strains isolated in Portugal were used. The characterization of these viruses is described in Boinas et al., 2004 [35]. Both viruses are haemadsorbing and pathogenic. Tomar 87 (henceforth virus T) was obtained from an ASF outbreak in 1987 in Tomar, a region north of the Tagus. This virus strain was passaged twice in pigs and once in *O*. *erraticus* ticks. OUR T88/1 (henceforth virus H) was obtained from ticks collected from a farm in the South of Portugal and was passaged once in a pig.

### Methods of exposure and virus titres of inoculum

Ticks were exposed to ASFV by feeding either on viraemic pigs or on artificial membranes (475 ticks, 95.2% of the total tested), or by being inoculated with virus suspensions (24 ticks, 4.8% of the total tested) (Table 1).

**Table 1:** Number of ticks exposed to ASFV summarized by method of exposure, virus titre and virus strain.

Method of exposure	Titres used	Virus	Number of ticks
**Pig feeding (PF)**	High (6.95)	T	26
		H	33
**Membrane feeding (MF)**	High (5.75)	T	92
	Low (3.0;5.0)		139
	High (5.75)	H	51
	Low (2.30;4.30)		134
**Total (PF and MF)**			475
**Inoculation (IN)**	High (5.75)	T	3
	Low (3.0)		10
	High (5.75)	H	1
	Low (3.0)		10
**Total (IN)**			24

Legend: T = virus T87; H = virus OUR T88/1

Six different titres of virus ranging from 2.3 to 6.95 log_10_HAD_50_/ml were used for exposure. Virus titres used in this study were classified in two groups, as ‘high’ or ‘low’, using 5.75 log_10_HAD_50_/ml as threshold value.

Two pigs were infected by intra-muscular inoculation; one with the virus strain T and the other with the virus strain H. The ticks were fed on the pigs, after anaesthetizing them, when their body temperature rose above 40°C (this occurred on the 4^th^ day post inoculation for Tomar 87 and on the 5^th^ day for OUR T88/1). The viraemia in both pigs was 10^6.95^ HAD_50_/ml [18].

Ticks were exposed by membrane feeding (MF) on viraemic blood diluted with PBM medium. The method described by Osborne and Mellor (1985) [36] for artificial feeding of blood to *O*. *moubata* complex using a silicone membrane was adapted to the "in vitro" infection of *O*. *erraticus sensu stricto* ticks with ASFV using a Parafilm membrane ("M", American National Can, Greenwich, CT.06836, USA) [18].

In order to make inferences regarding the potential existence of a gut barrier in *O*. *erraticus sensu stricto* ticks for these viruses, virus suspensions were inoculated directly into the haemocoel, thus bypassing the gut.

Ticks were anaesthetized with CO_2_ gas and transferred to a turntable under a dissecting microscope with a constant stream of CO_2_ gas to maintain anesthesia. The apparatus used for inoculation of the ticks has been described previously for *O*. *moubata* complex [37]. Volumes of the suspension inoculated varied from 1 μl for small nymphs to 5 μl for adults.

### Assay for infectious virus in *O*. *erraticus sensu stricto* ticks

For virus assay, ticks were surface sterilized with a 10% hypochlorite solution, washed with PBM diluent and homogenized individually using Tenbroek tissue grinders. Virus isolation and titration were performed by using a haemadsorption assay [38] by inoculating limiting dilutions of tick homogenates on pig bone marrow (PBM) cells. Undiluted and ten-fold dilutions of the inoculums were distributed between 3 tubes containing PBM culture.

The virus titres were calculated by the method of Reed and Muench (1938) [39] and expressed as 50% haemadsorbing doses (HAD_50_) per tick.

The period under analysis ranged from day 39 to day 117 post exposure to ASFV. Day 0 was not taken into account in our analysis and it was only used for monitoring the initial level of infection.

A tick was considered infected if it had a detectable titre of virus and if a titre equal to or higher than 10^4^ HAD_50_/tick was found, the tick was considered to have a high likelihood of virus excretion (LVE) [30].

### Statistical analysis

R software was used for descriptive and inferential analyses of the data.

Given some groups had too few observations to perform a Chi-Squared test, Fisher’s exact test was used to test the statistical significance of the relationship between the two viruses and between tick stages and the three outcome variables in the study: infection, likelihood of virus excretion (determined on the basis of virus titre) and likelihood of virus excretion within the infected ticks.

A set of logistic regression models were developed to assess the effect of tick gender (in adult ticks)(dichotomous variable: male or female), route of infection (categorical variable: Inoculation (IN); Pig feeding (PF) or membrane feeding (MF), virus titre to which ticks were exposed (dichotomous variable (high or low), tick stage (dichotomous variable: Small nymphs or larger stages (Adults and 5^th^ nymphal stage)), and time (days) post exposure (as a numerical variable), on the likelihood of the three outcome variables.

Statistical significance was defined as p ≤ 0.05.

## Results

### Descriptive statistics and univariate analysis of infection rate and likelihood of virus excretion by route of exposure, virus titre, virus strain, life cycle stage and gender

#### Pig-fed ticks

The ticks that fed on infected pigs were exposed to high titres of virus due to the viraemia of 10^6.95^ HAD_50_/ml achieved in pigs. A total of 33 ticks (55.9% of 59) fed on pigs with high titres of virus H and 26 ticks (44.1% of 59) fed on pigs with high titres of virus T (Table 2). The overall infection rate in the pig feeding group was 83.1% (49/59).

**Table 2:** Proportion of ticks fed on pigs with high blood titres of viruses H and T that became infected and with high likelihood of virus excretion (LVE).

Virus	Tick Stage	Gender	Total	Infected (N) *	Infected (%)*	LVE (N)*	LVE (%)*
**H**	Adults	Female	1	1	100.0	0	0.0
		Male	8	8	100.0	3	37.5
	Total		9	9	100.0	3	33.3
	n5		20	19	95.0	7	35.0
	n4		1	1	100.0	1	100.0
	n3		1	1	100.0	1	100.0
	n2		1	0	0.0	0	0.0
	n1		1	0	0.0	0	0.0
**Total**			33	30	90.9	12	36.4
**T**	Adults	Female	1	1	100.0	1	100.0
		Male	3	3	100.0	1	33.3
	Total		4	4	100.0	2	50.0
	n5		11	10	90.9	2	18.2
	n4		3	1	33.3	0	0.0
	n3		3	2	66.7	0	0.0
	n2		2	2	100.0	0	0.0
	n1		3	0	0.0	0	0.0
**Total**			26	19	73.1	4	15.4
**Total**			59	49	83.1	16	27.1

*For the total study period, i.e. from 39 to 117 days post exposure (DPE).

There were no significant differences between the two virus strains—H and T—neither for probability of infection (p = 0.09), nor for the LVE (p = 0.09), nor for the LVE within the infected ticks (p = 0.22).

In this group there was a statistically significant difference between adults and n5 grouped together (A-n5), and the group of small nymphs (n4-n1) with respect to the probability of being infected after 39 DPE, with A-n5 having a higher probability of infection compared with n4-n1 (p<0.001, Odds Ratio = 22.03, 95% confidence intervals [3.49; 253.83]). However, this effect was not seen for LVE and LVE within the infected ticks (p = 0.2 and p = 1 respectively) (Table 3).

**Table 3:** Differences between tick stages, A-n5 versus small nymphs, for each outcome—infection, likelihood of virus excretion (LVE) and likelihood of virus excretion within infected ticks (LVE within infected ticks).

RoE	Titre	Total stage		Infection			LVE			LVE within infected ticks		
		A-n5	n4-n1	p	OR	95% C.I.	p	OR	95% C.I.	p	OR	95% C.I.
PF	High	44	15	<0.001	22.03	[3.49; 253.83]	0.20	2.98	[0.55; 30.81]	1	1.24	[0.18; 14.62]
MF	High	111	32	0.0024	3.71	[1.49; 10.01]	0.71	0.75	[0.17; 4.68]	0.12	0.28	[0.05; 2.08]
	Low	261	12	1	inf	[0.08; inf]	1	inf	[0.008; inf]	1	0	[0; inf]
IN	High	3	1	0.25	inf	[0.08; inf]	0.25	inf	[0.08; inf]	0.25	inf	[0.08; inf]
	Low	18	2	1	inf	[0.003; inf]	-*			-*		

*Only one tick became infected but not LVE.

Legend: RoE—Route of exposure; PF—pig feeding; MF—membrane feeding; IN—inoculation; OR—odds ratio; C.I.—confidence interval.

There was no effect of gender on infection, LVE or LVE within the infected ticks (see S2 Table).

#### Membrane-fed ticks

A total of 143 ticks fed on membranes with high titres of viruses H or T (34.4% of 416) and 273 ticks fed on membranes with low titres (65.6% of 416) of either virus (Table 4).

**Table 4:** Proportion of ticks fed on membranes infected with high and low virus titres of strains H and T that became infected and with likelihood of virus excretion (LVE).

Titre	Virus	Tick Stage	Gender	Total	Infected (N) *	Infected (%)*	LVE (N)*	LVE (%)*
**High**	**H**	Adults	Female	4	2	50.0	0	0.0
			Male	15	7	46.7	1	6.7
		Total		19	9	47.4	1	5.3
		n5		19	15	78.9	1	5.3
		n4		1	0	0.0	0	0.0
		n3		4	1	25.0	0	0.0
		n2		5	1	20.0	0	0.0
		n1		3	3	100.0	1	33.3
	**Total**			51	29	56.9	3	5.9
	**T**	Adults	Female	12	6	50.0	1	8.3
			Male	22	14	63.6	3	13.6
		Total		34	20	58.8	4	11.8
		n5		39	22	56.4	2	5.1
		n4		5	2	40.0	2	40.0
		n3		8	1	12.5	0	0.0
		n2		3	0	0.0	0	0.0
		n1		3	1	33.3	0	0.0
	**Total**			92	46	50.0	8	8.7
**Total**				143	75	52.4	11	7.7
**Low**	**H**	Adults	Female	46	0	0.0	0	0.0
			Male	41	1	2.4	1	2.4
		Total		87	1	1.1	1	1.1
		n5		39	1	2.6	0	0.0
		n4		2	0	0.0	0	0.0
		n3		3	0	0.0	0	0.0
		n2		3	0	0.0	0	0.0
		n1		0	0	0.0	0	0.0
	**Total**			134	2	1.5	1	0.7
	**T**	Adults	Female	45	4	8.9	0	0.0
			Male	46	3	6.5	1	2.2
		Total		91	7	7.7	1	1.1
		n5		44	0	0.0	0	0.0
		n4		0	0	0.0	0	0.0
		n3		3	0	0.0	0	0.0
		n2		1	0	0.0	0	0.0
		n1		0	0	0.0	0	0.0
	**Total**			139	7	5.0	1	0.7
**Total**				273	9	3.3	2	0.7
**Total**				416	84	20.2	13	3.1

* For the total study period, i.e. from 39 to 117 days post-exposure.

The infection rate of ticks fed on high titre viruses was 52.4% (75/143) while for ticks fed on bloodmeals with low titre viruses it was 3.3% (9/273) (Table 4).

In both the high and low virus titre exposure groups, there was no statistically significant difference between virus H and T, neither for infection (p = 0.49 in the group that received high titres and p = 0.17 in the group that received low titres), nor for LVE (p = 0.75 and p = 1 in the high and low titre groups respectively), nor for LVE within the infected ticks (in the group that received high titres p = 0.51 and in the group that received low titres p = 0.42).

In the group of ticks that fed on membranes with high titres of viruses H or T, there were statistically significant differences between tick stages regarding to the probability of infection. Adults and n5 are more likely to be infected after 39 DPE than small nymphs (n4-n1) (p = 0.0024, Odds Ratio = 3.71, 95% confidence interval [1.49; 10.01]) (Table 3).

In membrane-fed ticks exposed to high titres of viruses H or T, there was no association between tick stage and LVE (p = 0.71 for LVE and p = 0.12 for LVE within the infected ticks) (Table 3).

In the group of ticks that fed via membranes on low titres of viruses H or T, there was no statistically significant difference between tick stages, for infection, for LVE or for LVE within the infected ticks (p = 1 for all outcomes) (Table 3).

There was also no effect of gender on infection, LVE or LVE within the infected ticks (see S2 Table).

#### Inoculated ticks

Sixty seven ticks were subjected to inoculation of virus, but only 24 were available for analysis 39 days post inoculation, which represents an overall mortality rate of 64.2%. For ticks inoculated with high titres the mortality rate was 88% and for ticks inoculated with low titres, 39.4%. In this way, only 4 ticks (16.7% of 24) inoculated with high titres of viruses H or T, and 20 ticks (83,3% of 24) inoculated with low titres of viruses H or T were available for analysis 39 days post inoculation (Table 5).

**Table 5:** Proportion of ticks inoculated with high and low titres of viruses H and T that became infected and with likelihood of virus excretion (LVE).

Titre	Virus	Tick Stage	Gender	Total	Infected (N)*	Infected (%)*	LVE (N)*	LVE (%)*
**High**	**H**	Adult	Male	1	1	100.0	1	100.0
	**Total**			1	1	100.0	1	100.0
	**T**	Adult	Male	1	1	100.0	1	100.0
		n5		1	1	100.0	1	100.0
		n4		1	0	0.0	0	0.0
	**Total**			3	2	66.7	2	66.7
**Total**				4	3	75.0	3	75.0
**Low**	**H**	Adult	Female	4	0	0.0	0	0.0
			Male	2	1	50.0	0	0.0
			Total	6	1	16.7	0	0.0
		n5		3	0	0.0	0	0.0
		n4		1	0	0.0	0	0.0
	**Total**			10	1	10.0	0	0.0
	**T**	Adult	Male	2	0	0.0	0	0.0
		n5		7	0	0.0	0	0.0
		n3		1	0	0.0	0	0.0
	**Total**			10	0	0.0	0	0.0
**Total**				20	1	5.0	0	0.0
**Total**				24	4	16.7	3	12.5

* For the total study period, i.e. from 39 to 117 days post-exposure

In both inoculated groups, high and low titres of virus, there was no significant statistical difference between the viruses H and T, neither for infection (p = 1), nor for LVE (p = 1), nor for LVE within the infected ticks (p = 1).

Also, as shown in Table 3, in the group of ticks inoculated with high virus titre, there were no differences between tick stages with respect to infection, LVE and LVE within the infected ticks.

There was also no effect of gender on infection, LVE or LVE within the infected ticks (see S2 Table).

### Multivariate analysis for effect of route of exposure, titre of exposure, tick stage (adults&N5 vs small nymphs) and DPE on infection, LVE and LVE within the infected ticks

Results of the univariate analyses were taken into account when grouping the six tick stages in adults and n5 (A-n5) and small nymphs (n4-n1).

The first model (A) compares membrane feeding to inoculation at high and low virus titres, the second model (B) compares all three routes of exposure but at high titres only, while a third model (C) makes use of all available data for the assessment of the statistical significance and magnitude of effects of the factors potentially affecting the infection and the likelihood of virus excretion.

The three models are consistent both in terms of statistical significance of analyzed variables and with respect to the estimated magnitude of effects (see in S3, S4 and S5 Tables, the adjusted values for odds ratios of variables with statistical significance).

In model A (Table 6), initial titre of virus exposure, tick stage and DPE have an influence on the likelihood of infection of ticks. Low titres of virus reduce infection probability and small nymphs are less likely to become infected than adults and n5. Also, the probability of infection decreases over time.

**Table 6:** Logistic regression model A—Effect of route of exposure (Inoculation versus Membrane feeding) and titre of exposure (high versus low titres), controlling for tick stage and days post exposure, in infection (n = 440), likelihood of virus excretion (LVE) (n = 440) and likelihood of virus excretion within the infected ticks (LVE within the infected ticks) (n = 88).

	Infection			LVE			LVE within infected ticks		
Variable	Value	P	OR C.I. 95%	Value	p	OR C.I. 95%	Value	p	OR C.I. 95%
Intercept	2.578	0.007	13.17 [1.96; 82.84]	-1.069	0.422	0.34 [0.02; 4.45]	-1.266	0.464	0.28 [0.009; 11.53]
RoE (MF)	-0.654	0.371	0.52 [0.13; 2.40]	-2.590	0.003	0.08 [0.01; 0.44]	-2.844	0.024	0.06 [0.003; 0.57]
Titre (Low)	-4.004	< 2e-^16^	0.02 [0.008; 0.04]	-2.964	0.001	0.05 [0.007; 0.23]	0.427	0.670	1.53 [0.15; 9.44]
Stage (Sm)	-1.473	0.001	0.23 [0.09; 0.53]	-0.156	0.822	0.86 [0.18; 2.98]	1.504	0.081	4.50 [0.76; 25.18]
DPE	-0.018	0.017	0.98 [0.97; 1.0]	0.015	0.233	1.02 [0.99; 1.04]	0.026	0.059	1.03 [1.0; 1.06]
Null dev.	440.35			137.46			83.45		
Res dev.	271.19			107.64			71.12		

Legend: RoE—Route of exposure; (MF)—membrane feeding; (Low)—low titre of virus exposure; (Sm)—small nymphal stages, n4-n1; DPE—days post exposure; Null dev.—null deviance; Res dev.—residual deviance; OR—Odds Ratio; C.I.—confidence interval.

When it comes to LVE, the effect of tick stage disappears. The variables that influence the likelihood of virus excretion are the route of infection (ticks that fed on membranes have a lower likelihood of virus excretion than ticks that were inoculated) and the initial titre has a marked influence on LVE (low titres are negatively associated with LVE).

For the likelihood of virus excretion within the infected ticks, the membrane feeding route is less likely to result in LVE in infected ticks while the initial titre has no effect. The variable time (days post exposure) has a near-significant positive effect (p = 0.06) on LVE of ticks given they are infected.

In model B (Table 7), it can be seen that infection of ticks is influenced by tick stage (with the small nymphs having a lower rate of infection than adults and n5) and by DPE, with the probability of infection decreasing over time. Comparing the three routes of virus exposure, membrane-fed ticks have a significantly lower rate of infection when compared with pig-fed ticks, but there are no statistically significant differences between pig-fed ticks and inoculated ticks in any of the outcomes.

**Table 7:** Logistic regression model B—Effect of route of exposure (Pig feeding versus inoculation and membrane feeding) with high titres of virus, controlling for tick stage and days post exposure, in infection (n = 206), likelihood of virus excretion (LVE) (n = 206) and likelihood of virus excretion within the infected ticks (LVE within the infected ticks) (n = 127).

	Infection			LVE			LVE within infected ticks		
Variable	Value	p	OR C.I. 95%	Value	p	OR C.I. 95%	Value	p	OR C.I. 95%
Intercept	3.436	1.33e^-06^	31 [8.14; 133.73]	-2.409	0.012	0.09 [0.01; 0.55]	-2.757	0.007	6.35e^-2^ [7.64e-3; 0.44]
RoE (MF)	-1.596	0.0003	0.20 [0.08; 0.47]	-1.937	0.0002	0.14 [0.05; 0.38]	-1.389	0.007	2.49e^-1^ [8.54e^-2^; 0.66]
RoE (IN)	-0.472	0.706	0.64 [0.06; 14.0]	2.066	0.108	7.90 [0.76; 187.90]	16.963	0.990	2.33e^+7^ [2.35e^-67^; NA]
Stage (Sm)	-1.871	3.87e^-06^	0.15 [0.07; 0.33]	-0.662	0.231	0.52 [0.16; 1.42]	0.551	0.384	1.74 [4.69e^-1^; 5.86]
DPE	-0.016	0.038	0.98 [0.97; 1.0]	0.022	0.083	1.02 [1.0; 1.05]	0.027	0.043	1.03 [1.0; 1.06]
Null dev.	274.29			171.00			138.86		
Res dev.	226.70			146.42			119.31		

Legend: RoE—Route of exposure; (MF)—membrane feeding; (IN)—inoculation; (Sm)—small nymphal stages, n4-n1; DPE—days post exposure; Null dev.—null deviance; Res dev.—residual deviance; OR—Odds Ratio; C.I.—confidence interval.

Both models (A and B) estimate a very similar rate of decay of infection (Tables 6 and 7).

The route of exposure is the only variable that has an influence on LVE by ticks (with membrane feeding having a poorer performance than pig feeding and inoculation of ticks). For the LVE within the infected ticks, membrane feeding remains the route of virus exposure leading to a significantly lower LVE and the time (days) post virus exposure had a statistically significant (p = 0.04) positive effect, with the LVE increasing as DPE increased.

Model C supports all the previous findings but is more comprehensive than models A or B. Its explained deviance of 47% and 27% for infection and LVE respectively, is better than that of models A or B.

The determining variables for ‘infection’ are route of virus exposure, titre, tick stage and DPE. Membrane feeding is the route resulting in the lowest probability of infection, which declines with days post exposure, and small nymphs have a lower likelihood of becoming infected than adults and n5.

With respect to the LVE, route of exposure and titre maintain significance while stage and DPE lose significance. This means that, for LVE, membrane feeding is significantly inferior to pig feeding and inoculation routes, low titres are less efficient than high titres, and there is no effect of tick stage or time post exposure, although LVE seems to rise as days post exposure increase.

In the subset of infected ticks this effect reaches statistical significance, with LVE within the infected ticks showing a statistically significant increase over time. Effect of route of exposure is maintained, but titre of exposure and stage are not significant for LVE within infected ticks (Table 8).

**Table 8:** Logistic regression model C—Effect of route of exposure (Pig feeding versus inoculation and membrane feeding) and titre of exposure (high and low titres), controlling for tick stage and days post exposure, in infection (n = 499), likelihood of virus excretion (LVE) (n = 499) and likelihood of virus excretion within the infected ticks (LVE within the infected ticks) (n = 137).

	Infection			LVE			LVE within infected ticks		
Variable	Value	p	OR C.I. 95%	Value	P	OR C.I. 95%	Value	p	OR C.I. 95%
Intercept	3.468	5.55e^-07^	32.09 [8.68; 132.78]	-2.217	0.012	0.11 [0.02; 0.59]	-2.626	0.006	0.07 [0.01; 0.45]
RoE (MF)	-1.582	0.0004	0.21 [0.08; 0.47]	-1.786	0.0003	0.17 [0.06; 0.43]	-1.279	0.01	0.28 [0.1; 0.71]
RoE (IN)	-0.896	0.281	0.41 [0.07; 1.97]	0.869	0.352	2.38 [0.35; 15.61]	1.441	0.262	4.23 [0.40; 98.55]
Titre (Low)	-4.058	< 2e^-16^	0.02 [0.008; 0.04]	-2.971	0.0006	0.05 [0.007; 0.23]	0.277	0.778	1.32 [0.14; 7.60]
Stage (Sm)	-1.877	2.98e-^06^	0.15 [0.07; 0.33]	-0.640	0.234	0.53 [0.17; 1.41]	0.513	0.413	1.70 [0.46; 5.55]
DPE	-0.016	0.026	0.98 [0.97; 1.0]	0.019	0.098	1.02 [1.0; 1.04]	0.025	0.042	1.03 [1.0; 1.05]
Null dev.	586.56			237.70			148.94		
Res dev.	312.50			173.84			133.74		

Legend: RoE—Route of exposure; (MF)—membrane feeding; (IN)—inoculation; Low—low titre of virus exposure; (Sm)—small nymphal stages, n4-n1; DPE—days post exposure; Null dev.—null deviance; Res dev.—residual deviance; OR—Odds Ratio; C.I.—confidence interval.

**Fig 1:**
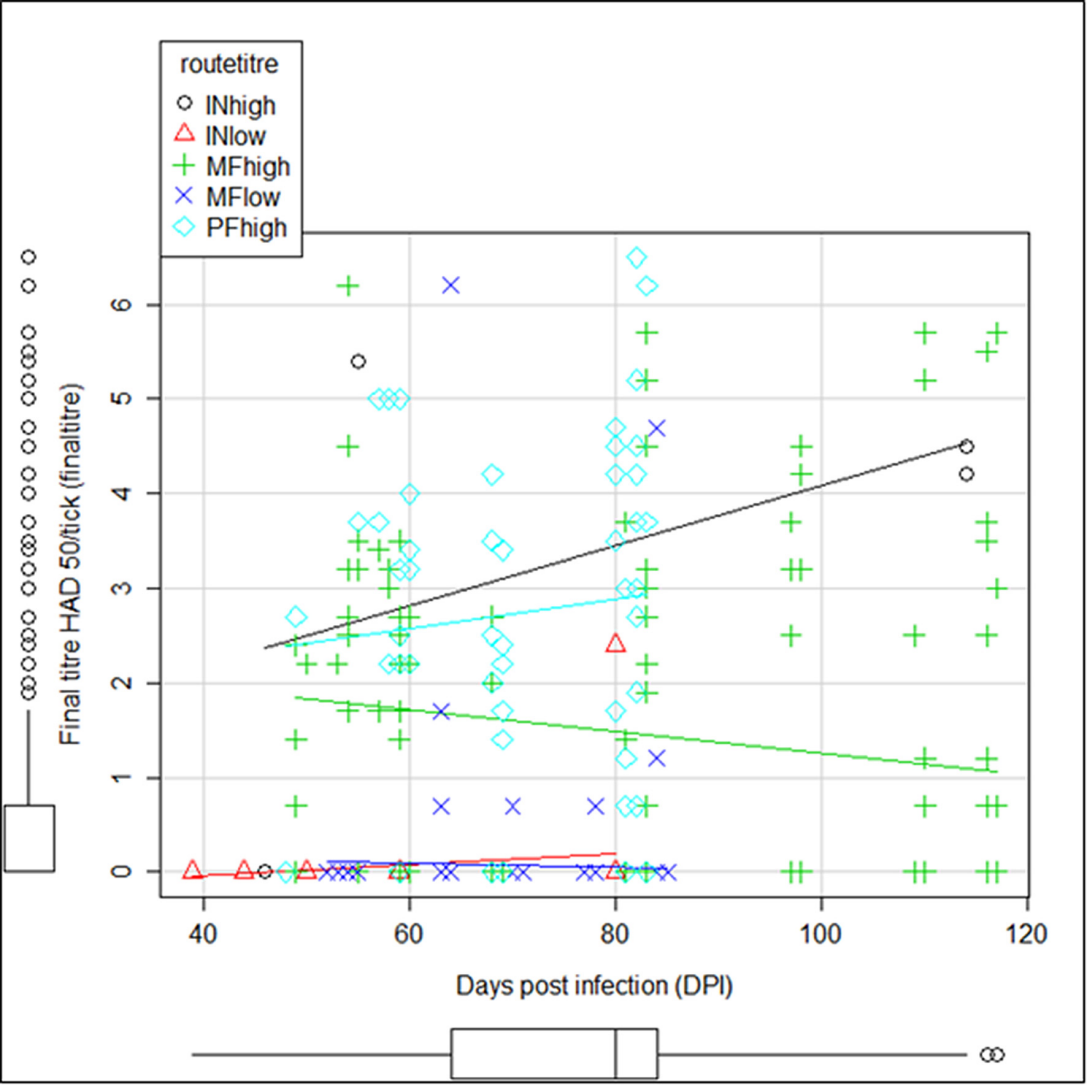
Scatterplot showing the variation of the final titre of virus in ticks (HAD _50_/Tick) between 39 days post infection and the end of the experiment per group—ticks inoculated with high and low titres of virus (INhigh and INlow); ticks that fed on membranes infected with high and low titres of virus (MFhigh and MFlow) and ticks that fed on pigs infected with high titres of virus (PFhigh).

Fig 1 shows the variation of the final titre of virus in the ticks, after 39 DPE until the end of the experiment. Most ticks that were exposed to low titres of virus, by inoculation or by membrane feeding, did not become infected. In the few ticks that became infected, the virus achieved a lower titre in the tick.

In ticks that were exposed to high titres of virus, by the three routes, the majority of ticks became infected and some developed virus titres regarded as sufficient for virus excretion, i.e. higher or equal to 10^4^HAD_50_/tick.

In ticks membrane fed with high titres, ticks became infected, and in some cases reached titres potentially indicative of virus excretion, but there was a downward trend in the final titre by tick over time. In contrast, in ticks exposed to high titres either by pig feeding or inoculation routes, there was an upward trend over time, regarding final titres by tick, meaning that the virus titres isolated from ticks later in time, at the end of the experiment, were higher than the titres isolated in the first days post exposure. The highest variation occurred in the group of ticks inoculated with high titres.

## Discussion

The population of ticks used in this experiment consisted of a high number of adults and ticks in the last nymphal stage with only a few specimens in the early nymphal stages. We have performed a random sampling and we believe that this reflects the population of ticks that is usually infesting pig farms.

The analyses for infection and LVE comprised all exposed ticks, with LVE being the epidemiologically more meaningful outcome due to the likeliness of ASFV transmission to susceptible hosts when the ticks feed. The analysis of LVE within infected ticks was conducted to further differentiate the factors that influence the likelihood of infection and of LVE.

The criteria followed for the classification of a tick as being actively infected or as having a likelihood for virus excretion are based on previous studies with *O*. *erraticus sensu stricto* ticks and *O*. *moubata* complex. Basto and co-workers (2006) showed that in experimental infections of *O*. *erraticus sensu stricto* ticks, titres of ASFV at four weeks post experimental feeding were higher than the initial amount of virus ingested, indicating that after four weeks, virus replicates [19]. Rowlands et al. (2009) and Diaz et al. (2012) further found that it takes three to four weeks for *O*. *erraticus sensu stricto* ticks to completely digest and clear ingested blood, thus the viruses isolated after this period are due exclusively to viral replication [3,40].

Assuming that recovery of virus from day 30 post-exposure onwards was the result of an active infection with viral replication and that our ticks were tested after day 39, this was our starting point for the experiment. In this way we attain a “security interval” to a total clearance of ingested virus.

Based on observations of Wilkinson and Mellor (not published) cited by Haresnape and Wilkinson (1989) in which ticks of the *O*. *moubata* complex that achieved a titre of 10^4^ HAD_50_/tick were more likely to excrete ASFV when feeding [30], we classified a tick as likely to excrete virus if the titre was ≥ 10^4^ HAD_50_.

As not all combinations of factors possibly influencing infection and LVE could be experimentally tested (e.g. exposure via pig feeding exclusively occurred with ‘high’ titres) and as the number of observations for some combinations was very low (e.g. high tick mortality when inoculating ticks), a number of multivariate analyses were performed to test for the statistical significance of the putative risk factors and estimate their magnitude while controlling for confounding effects.

Model A compares the two experimental routes of virus exposure, membrane feeding and inoculation of virus, and tests the effect of the initial titre, tick stage and days post virus exposure. In model B we removed the possible effect of the initial titre and only assessed ticks that were exposed to high initial titres of virus, by all routes of exposure. Model C is a validation of the previous results, combining all variables.

Hereafter we characterize the effect of the several factors included in this analysis.

### ASF virus strains

Two different haemadsorbing viruses, both virulent in pigs, were used, virus H isolated from ticks and virus T, isolated from pigs [35]. We found no differences between the two viruses in the probability of infection, LVE, and LVE within the infected ticks, suggesting that *O*. *erraticus sensu stricto* ticks can be equally competent for two different haemadsorbing ASF viruses of different host origins. This contrasts to previous studies with ticks from *O*. *moubata* complex in which remarkable differences were found between African virus isolates of tick and of pig origins, in their ability to infect tick populations [11]. Nevertheless, these differences could be due to the high genetic diversity present in sub-Saharan Africa where 22 genotypes have been recorded [41,42], in contrast to the similarity between European field strains which all belong to a single genotype (I) and are linked to the virus introduced to Portugal in 1960 and sharing a common ancestry [35,43]. The fact that virus H was isolated from ticks doesn’t necessary mean that this virus strain is highly adapted to ticks, as it may have infected ticks occasionally when they were feeding on pigs. Additionally, there is a need for a long history of coevolution for tick-virus adaptation to occur [9], and we weren’t able to estimate if that period was long enough for coevolution to occur.

### Tick stage

Since day 39 post exposure, the group of adults and n5 that fed on pigs or on membranes with high titres had a significantly higher likelihood of being classified as infected than small stage nymphs (85.7% vs 14.3%).

These differences can be explained by the higher volumes that large stages are able to ingest, and therefore the titre of virus which is maintained in the tick after the bloodmeal digestion.

This was also observed in the comparison of all tick stages on the day of oral exposure and, although no difference was observed in the rate of virus recovery, the larger stages (n5 and adults) had higher titres of virus than small nymphs [18].

Taking into account the differences found between the tick stages, for the multivariate analyses we grouped the ticks in “A-n5” and “n4-n1”. In all three models, tick stage had a significant effect on the likelihood of infection, with the small nymphs having a much lower likelihood of infection than larger stages (OR = 0.15), which corroborated our previous hypothesis.

Other studies have also reported a more prolonged infection with ASFV for adults and n5 than for small nymphs [18, 19].

An effect of ‘tick stage’ was however not maintained in any of the models for LVE and LVE within the infected ticks. Although no statistical differences for LVE were observed, a higher number of A-n5 had a high LVE in comparison with n4-n1, and thus the inability to demonstrate statistical difference could be due to the small number of ticks with titres higher than 10^4^ HAD_50_/tick in both groups.

### Gender of adult ticks

Our results are indicative that gender of exposed adult ticks does not play a role in either the likelihood of infection or in the likelihood of virus excretion.

Although females can ingest, on average, 3 times more blood than males [18], the chances of them becoming infected or competent are the same. This suggests that once a threshold value is reached, the number of virus particles exceeding the amount necessary to cause infection become irrelevant.

### Titre at exposure

Taking into account the level of viraemia in the infected pigs (6.95 log_10_HAD_50_/ml), and the highest titre level used to feed ticks through artificial routes (5.75 log_10_HAD_50_/ml) we defined two groups: the ‘high’ group, which included ticks fed with titres of 10^6.95^ HAD_50_/ml and 10^5.75^HAD_50_/ml, and the ‘low’ group, which consisted of ticks fed with titres of 10^2.3^; 10^3^; 10^4.3^; 10^5^ HAD_50_/ml. The purpose of have these two groups is that ‘high’ titres would relate to acute infections while ‘low’ titres would be associated with sub-acute and chronic infections [3, 19, 35, 44].

Ticks that were exposed to low titres of virus had a much lower likelihood of infection (OR ≈ 0.02) and LVE than those exposed to high titre bloodmeals (OR ≈ 0.05). However, the effect of the titre of exposure disappeared when analyzing LVE within the infected ticks (models A and C). This suggests that high titres are important for the establishment of infection but that once infected, LVE can develop independently from the initial inoculum.

A hypothesis for this could relate to the finding referred to by previous pathogenesis studies, suggesting that ASFV follows the well-described virus-tick pathways: ingestion of bloodmeal, replication of the virus in the midgut, entering the haemocoel and infecting the major secretory glands, the coxal and salivary glands [45], which can also act as a barrier for infection [46]. This would explain the infection and the concurrent absence of high titres as viruses wouldn’t replicate in the target organs.

Nevertheless, in some cases viruses could not even cross the gut barrier being unable to multiply within the tick, as shown by Kleiboeker (1999) [31] using *O*. *porcinus* ticks and ASFV strain Malawi Lil 20/1 with titres reaching 10^7^ HAD_50_/ml.

In our experiment, infection occurred occasionally in ticks exposed to low initial titres. The existence of a discontinuity in the digestive mucosa, characterized by the presence of lesions or pores between the gut cells formed during the first phase of digestion, known as "leaky gut" [47], allows the passage of the virus directly to the haemocoel without entering the gut cells, thus by-passing the "gut barrier". The contents of the gut have been shown to occasionally leak into the haemocoel during or shortly after feeding [31]. This could explain our results, mimicking the effect of inoculation.

Virulent viruses frequently cause high virus titres (10^8−9^ HAD_50_/ml) and acute disease in pigs, with death occurring 6–8 days post-infection [48]. In the less acute infections, viraemia is lower and longer [49].

Our results suggest that in cases of acute disease, the probability of infection and LVE by ticks will be significantly higher than when pigs are affected by a less virulent strain.

### Route of exposure

The pig feeding route was considered as the reference for comparing the two other infection routes, as it is the route by which ticks become infected in field conditions.

Pigs experimentally infected with ASFV achieved a viraemia of 10^6.95^ HAD _50_/ml and the group of ticks that fed on pigs was exposed to this titre, which corresponds to the peak viraemia of an acute infection [35].

Of the ticks fed on highly viraemic pigs, 83.1%, were classified as infected 39 days post exposure while only 23.1% developed virus titres regarded as sufficient for virus excretion.

We did not find in the literature scientific evidence of the existence of different proteins that could influence the outcome of the tick infection if feeding with diluted blood of acutely infected pigs versus chronically infected pigs. Studies on chronically ASF-infected pigs reported only the presence of hyperglobulinemia and did not suggest the presence of different protein expression patterns [50]. In this way, membrane feeding was used as a way of replicating the natural decrease of viraemia in subacute /chronic cases, by feeding ticks on dilutions of infected pig blood.

Inoculation of virus into the tick haemocoel, bypassing the gut, and subsequent virus replication was used to evaluate the presence of a gut barrier. There was a high mortality rate for inoculated ticks, which could be due to the damage caused by inoculation to tick tissues and organs.

In our experiment we have not analyzed ticks that died following inoculation, because we could not guarantee that the natural degradation of the dead ticks wouldn’t interfere with the virus titration possibly causing inactivation of the virus.

Rate of mortality of *O*. *erraticus sensu stricto* ticks following infection has been previously evaluated, only for orally infected ticks. Hess et al. (1989) and Endris & Hess (1994) showed that mortality rate of *O*.*erraticus sensu stricto* ticks infected with ASFV was considerably higher than in uninfected ticks [28,32]. The most probable cause of death suggested was gut rupture that could be due to a massive virus infection of gut cells, which weakens the gut wall, making it prone to rupture, particularly when taking a large bloodmeal [28].

The infection of ticks is influenced by the route of exposure, with membrane-fed ticks having a lower rate of infection compared with pig-fed ticks. Tick LVE, and LVE within infected ticks, is also influenced by the route of exposure with membrane-fed ticks having a lower LVE than pig-fed and inoculated ticks, even though they were membrane fed with high titre of virus. This outcome is expected, as the pig fed group had a slightly higher viraemia (one log of difference between high titres) and inoculation implies that the gut barrier is overcome.

Exposure to low virus titres via membrane feeding resulted in significantly lower rates of infection and LVE, 20.2% and 3.1% respectively, however, indicating that ticks can also become infected on pigs that have overcome the acute phase of infection.

Some of the membrane fed ticks had high titres of virus during the course of the experiment, and this could be due to individual variations or the presence of a "leaky gut".

Although no statistical difference was found between inoculated and pig-fed ticks, a titre ≥ 10^4.0^ HAD _50_/tick after 39 days post virus exposure was more likely to occur in inoculated ticks than in those exposed orally (feeding on pigs and on membranes). These results help to corroborate that a “gut barrier” exists in ticks, which prevents access of the virus present in the tick intestine to the haemolymph and to the other tick organs where the virus replicates, but it seems that when the threshold is surpassed, this gut barrier is less efficient.

### Days post exposure (DPE)

The variable days post exposure (DPE) was considered as a numerical variable in the multivariate logistic model. The implicit assumption is that the log odds ratio of being infected (or Likelihood of virus excretion) varies linearly with DPE. So, the model analyses DPE as a trend and searches for the effect on the response variable (infection or LVE or LVE within infected ticks). The time post virus exposure has a negative effect on the likelihood of infection, in the three models, which means that the proportion of ticks classified as infected decreases over time. For LVE within the infected ticks, the effect of the DPE becomes positive, suggesting that in ticks infected with high titres, virus titres are maintained for a long period, corroborating the previous reports that identify *O*. *erraticus sensu stricto* ticks has reservoirs of ASFV in the medium and long term period, as they are responsible for the persistence of infection in the field for periods of up to 5 years [2]. In contrast, infection of *O*. *erraticus sensu stricto* ticks with low titres of ASFV does not allow the maintenance of virus in ticks.

It seems that two periods of virus clearance exist in ticks; one until 30 days post infection in which the ingested virus present in the gut, that does not pass the mid-gut barrier, is eliminated and another coinciding with a post-30-day period, which is related with the clearance of the virus that replicated within the tick organs. Only ticks with a rate of virus replication higher than virus clearance will become persistently infected and only these would be able to excrete virus. In addition to the variables assessed in our study, this is likely also influenced by the biological variability of ticks.

The technique for virus titration in the exposed ticks detected low titres of virus (10^0.7^HAD/tick), which can correspond to trace amounts that can be influenced by the sensitivity of the assay and by the method of tick homogenization.

## Conclusions

This work is the result of a set of studies that aimed to understand the role of the *Ornithodoros erraticus sensu stricto* ticks in the epidemiology of ASFV, particularly the identification of factors that influence the dynamics of infection in ticks, and that can be important for future vectorial competence studies.

We demonstrate that *Ornithodoros erraticus sensu stricto* ticks can have high titres of two haemadsorbing ASF viruses from different origins, which can be indicative of a higher likelihood of virus excretion. The ability to excrete the virus is an important component of vectorial competence, so the results of this study suggest that *O*. *erraticus sensu stricto* can be a competent vector for virus OUR T88/1 and T87, particularly when feeding on highly viraemic pigs, a typical situation in ASF outbreaks. We have also proven that *O*. *erraticus sensu stricto* ticks, can become infected on pigs with lower titres of viruses OUR T88/1 and T87, which in the field correspond to animals that have sub-acute and or chronic forms: either by surviving the acute phase of infection or if they were infected with less virulent virus.

In a region affected by a new type of ASFV, as it is presently the situation in the Caucasus, Russian federation and eastern Europe [3] and due to the presence of *O*. *erraticus sensu stricto* ticks in the Trans-Caucasus region, it is important to investigate the local populations of *Ornithodoros* ticks as they could contribute to the persistence of infection in the area and compromise control measures. Additionally, the rapid spread of the disease in Europe is alarming and the possibility of the disease reaching countries where proven competent vectors exist, such as the Iberian Peninsula, must be seriously considered.

Regions with ASF outbreaks associated with acute disease in pigs need a strong tick surveillance and control because acute infection promotes higher rates of infection and competence of ticks, compared with chronic infections that probably do not play an important role in the epidemiology of the disease.

The higher infection rates and higher bloodmeal volumes of adults and large nymphal stages suggests that, in field surveys, the analysis of these stages provide the best chance of detecting virus infection [2,51]. This fact was also found for *Ornithodoros moubata* complex ticks. Plowright, (1977) and Thomson et al., (1983) reported the higher ASFV presence in adult tick pools compared to nymph tick pools [52,53].

In cases of high density of ticks or hyperparasitism in pig shelters, which is well documented in *O*. *erraticus sensu stricto* ticks [54] we can expect, based on the findings of this study, that infection rate and LVE by ticks can increase. The increase in the LVE in infected ticks over time stresses the importance of following current EU legislation guidelines for repopulation of pig shelters after six years of ASFV outbreaks [55].

Database on which this work was based will be made available (see S6 Table), to facilitate future studies investigating the acquisition of competence by vectors. Other investigations should be made using other vector species as well as other virus strains in order to study a wider range of tick-virus associations.

## Supporting Information

S1 Table Locations where *O*. *erraticus sensu stricto* ticks used in the experiment were collected. Name and geographic coordinates of the parish centroid.
(DOCX)

S2 Table Effect of the gender of adult ticks on infection, LVE and LVE within infected ticks. (DOCX)

S3 Table Logistic regression model A—Effect of route of exposure (Inoculation versus Membrane feeding) and titre of exposure (high versus low titres), controlling for tick stage and days post exposure, in infection (n = 440), LVE (n = 440) and LVE within the infected ticks (n = 88). Value of coefficients and odds ratio for the variables with statistical significance.
(DOCX)

S4 Table Logistic regression model B—Effect of route of exposure (Pig feeding versus inoculation and membrane feeding) with high titres of virus, controlling for tick stage and days post exposure, in infection (n = 206), LVE (n = 206) and LVE within the infected ticks (n = 127). Value of coefficients and odds ratio for the variables with statistical significance.
(DOCX)

S5 Table Logistic regression model C—Effect of route of exposure (Pig feeding versus inoculation and membrane feeding) and titre of exposure (high and low titres), controlling for tick stage and days post exposure, in infection (n = 499), LVE (n = 499) and LVE within the infected ticks (n = 137). Value of coefficients and odds ratio for the variables with statistical significance.
(DOCX)

S6 Table Dataset of all experiments performed to investigate the dynamics of infection of *Ornithodoros erraticus sensu stricto* ticks with two strains of African swine fever virus. (XLSX)
