# Supplementary material for: Experimental Infection of Ornithodoros erraticus sensu stricto with Two Portuguese African Swine Fever Virus Strains. Study of Factors Involved in the Dynamics of Infection in Ticks
Source: PLoS One. 2015 Sep 14;10(9):e0137718. doi: 10.1371/journal.pone.0137718 (PMC4569400; doi:10.1371/journal.pone.0137718)
Supplement: S1 Table — Name and geographic coordinates of the parish centroid. (DOCX) [file pone.0137718.s001.docx]

S1 Table: Locations where *O. erraticus sensu stricto* ticks used in the experiment were collected. Name and geographic coordinates of the parish centroid.

|  | **Geographic coordinates of the parish centroid** | |
| --- | --- | --- |
| **Parish** | **Latitude** | **Longitude** |
| ALQUEVA | 38.224020 | -7.5461121 |
| CASTRO VERDE | 37.707133 | -8.0801212 |
| MONSARAZ | 38.452305 | -7.3917820 |
| SANTO ANTONIO DAS AREIAS | 39.420066 | -7.3368714 |
| SAO MARCOS DA SERRA | 37.371552 | -8.3587106 |
| SANTANA DE CAMBAS | 37.649201 | -7.5385312 |
| CORTE DO PINTO | 37.713358 | -7.4807030 |
