# Supplementary material for: Experimental Infection of Ornithodoros erraticus sensu stricto with Two Portuguese African Swine Fever Virus Strains. Study of Factors Involved in the Dynamics of Infection in Ticks
Source: PLoS One. 2015 Sep 14;10(9):e0137718. doi: 10.1371/journal.pone.0137718 (PMC4569400; doi:10.1371/journal.pone.0137718)
Supplement: S2 Table — (DOCX) [file pone.0137718.s002.docx]

S2 Table: Effect of the gender of adult ticks in infection, competence and competence within infected ticks.

| **Route of exposure** | **Titre** |  | **Infection** | | | | **Competence** | | | **Competence within infected ticks** | |
| --- | --- | --- | --- | --- | --- | --- | --- | --- | --- | --- | --- |
|  |  |  | **Coefficient** | | | **p** | **Coefficient** | **p** | | **Coefficient** | **p** |
| **Pig feeding*** | **High** | Intercept | -2.46e^+01^ | | | 1 | -3.00e^-17^ | 1 | | -3.00e^-17^ | 1 |
|  |  | Gender (Male) | -1.89e^-14^ | | | 1 | -5.60e^-01^ | 0.72 | | -5.60e^-01^ | 0.72 |
|  |  | Null dev. | 0.00e^+00^ | | | | 17.32 | | | 17.32 | |
|  |  | Res dev. | 5.57e^-10^ | | | | 17.19 | | | 17.19 | |
|  |  | DfND | 12 | | | | 12 | | | 12 | |
| **Membrane feeding** | **High** | Intercept | 2.12e^-16^ | | 1 | | -2.71 | 0.01 | | -1.95 | 0.07 |
|  |  | Gender (Male) | 2.72e^-01^ | | 0.65 | | 0.60 | 0.61 | | 0.50 | 0.68 |
|  |  | Null dev. | 73.00 | | | | 33.12 | | | 26.66 | |
|  |  | Res dev. | 72.80 | | | | 32.83 | | | 26.48 | |
|  |  | DfND | 52 | | | | 52 | | | 28 | |
|  | **Low** | Intercept | -3.08 | 1.71e^-09^ | | | -21.57 | | 0.99 | -19.57 | 1 |
|  |  | Gender (Male) | 0.05 | 0.95 | | | 17.82 | | 0.99 | 19.57 | 1 |
|  |  | Null dev. | 65.27 | | | | 21.93 | | | 9.00 | |
|  |  | Res dev. | 65.27 | | | | 19.05 | | | 5.55 | |
|  |  | DfND | 177 | | | | 177 | | | 7 | |
| **Inoculation** | **High **** | - | - | | | | - | | | - | |
|  | **Low** | Intercept | -19.57 | | 1 | | -2.46e^+01^ | | 1 | -*** |  |
|  |  | Gender (Male) | 18.47 | | 1 | | -3.75e_-15_ | | 1 |  |  |
|  |  | Null dev. | 6.03 | | | | 0.00e^+00^ | | |  | |
|  |  | Res dev. | 4.50 | | | | 3.43e^-10^ | | |  | |
|  |  | DfND | 7 | | | | 7 | | |  | |

Legend: Null dev. – null deviance; Res dev. – residual deviance; DfND – degrees of freedom null deviance.

*In the pig feeding group all the 13 adults became infected.

**Only 2 adult males were inoculated with high titres of virus. Both became infected and competent.

*** Only one adult male became infected but not competent.
