# Supplementary material for: Experimental Infection of Ornithodoros erraticus sensu stricto with Two Portuguese African Swine Fever Virus Strains. Study of Factors Involved in the Dynamics of Infection in Ticks
Source: PLoS One. 2015 Sep 14;10(9):e0137718. doi: 10.1371/journal.pone.0137718 (PMC4569400; doi:10.1371/journal.pone.0137718)
Supplement: S3 Table — Value of coefficients and odds ratio for the variables with statistical significance. (DOCX) [file pone.0137718.s003.docx]

S3 Table: Logistic regression model A - Effect of route of exposure (Inoculation versus Membrane feeding) and titre of exposure (high versus low titres), controlling for tick stage and days post exposure, in infection (n=440), competence (n=440) and competence within the infected ticks (n=88). Value of coefficients and odds ratio for the variables with statistical significance.

|  | **Infection** | | | **Competence** | | | **Competence within infected ticks** | | |
| --- | --- | --- | --- | --- | --- | --- | --- | --- | --- |
| **Variable** | **Value** | **p** | **OR**  **C.I. 95%** | **Value** | **p** | **OR**  **C.I. 95%** | **Value** | **p** | **OR**  **C.I. 95%** |
| Intercept | 1.97 | 0.003 | 7.16  [1.99; 27.42] | 0.09 | 0.92 | 1.09  [0.20; 6.01] | 1.1 | 0.34 | 3  [0.38; 60.65] |
| RoE (MF) |  |  |  | -2.48 | 0.003 | 0.08  [0.02; 0.47] | -2.80 | 0.02 | 0.06  [0.003; 0.52] |
| Titre (Low) | -3.96 | < 2e^-16^ | 0.02  [0.008; 0.04] | -3.13 | 0.0002 | 0.04  [0.006; 0.18] |  |  |  |
| Stage (Sm) | -1.46 | 0.001 | 0.23  [0.09; 0.53] |  |  |  |  |  |  |
| DPE | -0.02 | 0.02 | 0.98  [0.97; 1.0] |  |  |  |  |  |  |
| Null dev. | 440.4 |  |  | 137.5 |  |  | 83.5 | 83.5 |  |
| Res dev. | 272.0 |  |  | 109.2 |  |  | 76.9 | 74.0 |  |

Legend: RoE – Route of exposure; (MF) – membrane feeding; (IN) - inoculation; Low – low titre of virus exposure; (Sm) – small nymph stages, n4-n1; DPE – days post exposure; Null dev. – null deviance; Res dev. – residual deviance; OR – Odds Ratio; C.I. – confidence interval.
