# Supplementary material for: Experimental Infection of Ornithodoros erraticus sensu stricto with Two Portuguese African Swine Fever Virus Strains. Study of Factors Involved in the Dynamics of Infection in Ticks
Source: PLoS One. 2015 Sep 14;10(9):e0137718. doi: 10.1371/journal.pone.0137718 (PMC4569400; doi:10.1371/journal.pone.0137718)
Supplement: S4 Table — Value of coefficients and odds ratio for the variables with statistical significance. (DOCX) [file pone.0137718.s004.docx]

S4 Table: Logistic regression model B - Effect of route of exposure (Pig feeding versus inoculation and membrane feeding) with high titres of virus, controlling for tick stage and days post exposure, in infection (n=206), competence (n=206) and competence within the infected ticks (n=127). Value of coefficients and odds ratio for the variables with statistical significance.

|  | **Infection** | | | **Competence** | | | **Competence within infected ticks** | | |
| --- | --- | --- | --- | --- | --- | --- | --- | --- | --- |
| **Variable** | **Value** | **p** | **OR**  **C.I. 95%** | **Value** | **p** | **OR**  **C.I. 95%** | **Value** | **p** | **OR**  **C.I. 95%** |
| Intercept | 3.44 | 1.33e^-6^ | 31  [8.14; 133.73] | -1.0 | 0.0007 | 0.37  [0.20; 0.65] | -2.71 | 0.009 | 6.66e^-2^  [7.85e^-3^; 0.47] |
| RoE (MF) | -1.60 | 0.0003 | 0.20  [0.08; 0.47] | -1.50 | 0.0005 | 0.22  [0.09; 0.51] | -1.42 | 0.006 | 2.41e^-1^  [8.20e^-2^; 0.64] |
| RoE (IN) | -0.47 | 0.71 | 0.64  [0.06; 14.0] | 2.10 | 0.080 | 8.06  [0.96; 169.68] | 16.87 | 0.99 | 2.13e^7^  [2.59e^-67^; NA] |
| Stage (Sm) | -1.87 | 3.87e^-6^ | 0.15  [0.07; 0.33] |  |  |  |  |  |  |
| DPE | -0.02 | 0.04 | 0.98  [0.97; 1.0] |  |  |  | 0.03 | 0.04 | 1.03  [1.0; 1.06] |
| Null dev. | 274.3 |  |  | 171.0 |  |  | 138.9 |  |  |
| Res dev. | 226.7 |  |  | 151.0 |  |  | 120.0 |  |  |

Legend: RoE – Route of exposure; (MF) – membrane feeding; (IN) - inoculation; Low – low titre of virus exposure; (Sm) – small nymph stages, n1-n4; DPE – days post exposure; Null dev. – null deviance; Res dev. – residual deviance; OR – Odds Ratio; C.I. – confidence interval.
