# Supplementary material for: Experimental Infection of Ornithodoros erraticus sensu stricto with Two Portuguese African Swine Fever Virus Strains. Study of Factors Involved in the Dynamics of Infection in Ticks
Source: PLoS One. 2015 Sep 14;10(9):e0137718. doi: 10.1371/journal.pone.0137718 (PMC4569400; doi:10.1371/journal.pone.0137718)
Supplement: S5 Table — Value of coefficients and odds ratio for the variables with statistical significance. (DOCX) [file pone.0137718.s005.docx]

S5 Table: Logistic regression model C - Effect of route of exposure (Pig feeding versus inoculation and membrane feeding) and titre of exposure (high and low titres), controlling for tick stage and days post exposure, in infection (n=499), competence (n=499) and competence within the infected ticks (n=137). Value of coefficients and odds ratio for the variables with statistical significance.

|  | **Infection** | | | **Competence** | | | **Competence within infected ticks** | | |
| --- | --- | --- | --- | --- | --- | --- | --- | --- | --- |
| **Variable** | **Value** | **p** | **OR**  **C.I. 95%** | **Value** | **p** | **OR**  **C.I. 95%** | **Value** | **p** | **OR**  **C.I. 95%** |
| Intercept | 3.47 | 5.55e-^07^ | 32.09  [8.68; 132.78] | -0.99 | 0.0007 | 0.37  [0.20; 0.65] | -2.53 | 0.007 | 0.08  [0.01; 0.48] |
| RoE (MF) | -1.58 | 0.0004 | 0.21  [0.08; 0.47] | -1.41 | 0.0008 | 0.24  [0.11; 0.55] | -1.28 | 0.007 | 0.28  [0.11; 0.69] |
| RoE (IN) | -0.90 | 0.28 | 0.41  [0.07; 1.97] | 1.08 | 0.22 | 2.93  [0.49; 17.65] | 1.44 | 0.25 | 4.22  [0.43; 93.99] |
| Titre (Low) | -4.06 | < 2e^-16^ | 0.02  [0.008; 0.04] | -3.13 | 0.0002 | 0.04  [0.006; 0.18] |  |  |  |
| Stage (Sm) | -1.88 | 2.98e^-06^ | 0.15  [0.07; 0.33] |  |  |  |  |  |  |
| DPE | -0.02 | 0.03 | 0.98  [0.97; 1.0] |  |  |  | 0.03 | 0.04 | 1.03  [1.0; 1.05] |
| Null dev. | 586.6 |  |  | 237.7 |  |  | 148.9 |  |  |
| Res dev. | 312.5 |  |  | 178.1 |  |  | 134.4 |  |  |

Legend: RoE – Route of exposure; (MF) – membrane feeding; (IN) - inoculation; Low – low titre of virus exposure; (Sm) – small nymph stages, n1-n4; DPE – days post exposure; Null dev. – null deviance; Res dev. – residual deviance; OR – Odds Ratio; C.I. – confidence interval.
